# Using the Gene Pulser MXcell Electroporation System to Transfect Primary Cells with High Efficiency

**DOI:** 10.3791/1662

**Published:** 2010-01-07

**Authors:** Adam M. McCoy, Michelle L. Collins, Luis A. Ugozzoli

**Affiliations:** Gene Expression Division, Bio-Rad Laboratories, Inc.

## Abstract

It is becoming increasingly apparent that electroporation is the most effective way to introduce plasmid DNA or siRNA into primary cells. The Gene Pulser MXcell electroporation system and Gene Pulser electroporation buffer (Bio-Rad) were specifically developed to easily transfect nucleic acids into mammalian cells and difficult-to-transfect cells, such as primary and stem cells. We will demonstrate how to perform a simple experiment to quickly identify the best electroporation conditions. We will demonstrate how to run several samples through a range of electroporation conditions so that an experiment can be conducted at the same time as optimization is performed. We will also show how optimal conditions identified using 96-well electroporation plates can be used with standard electroporation cuvettes, facilitating the switch from electroporation plates to electroporation cuvettes while maintaining the same electroporation efficiency. In the video, we will also discuss some of the key factors that can lead to the success or failure of electroporation experiments.

**Figure:**
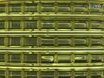


## Protocol

### 1) Cell preparation

When using adherent cells, it is necessary to trypsinize and collect the cells prior to electroporation.
To compare transfection among the four mouse embryonic fibroblast (MEF) cultures, representing three different passage numbers, perform the following on each flask.
Aspirate off cell culture media.
Add PBS to wash cells.
Remove PBS; add sufficient trypsin to cover cells, and wait a few minutes to allow trypsin to detach cells.
Check flasks with a microscope to verify the condition of the cells, smack flask to detach cells, and then check flask again to make sure cells are all detached.
Wait additional time and repeat if necessary.
Once all cells are detached, add serum containing media to neutralize trypsin.
Transfer cells to a centrifuge tube, and pellet cells by centrifugation (rcf = 300 x g).
Remove supernatant and resuspend cells in a known volume of PBS.
Count cells.
Transfer to a new tube the appropriate volume of cell suspension to provide the required number of cells for experiments (you will need 150 μL of cells per well at a density of 1 x 10^6^ cells/mL).
Centrifuge cells.
Remove supernatant and resuspend cells in the appropriate volume of Gene Pulser electroporation buffer to achieve a cell density of 1 x 10^6^ cells/mL.
Add 20 μg of plasmid per mL of cell suspension and mix gently.

### 2) Electroporation vessel setup and electroporation

#### Plate setup and electroporation

Plug plate chamber into the power module of the Gene Pulser MXcell electroporation system.
Pipette 150 μL cell mixture or buffer into the wells of a 96-well electroporation plate.
Put plate in plate chamber and pulse.
Remove plate from chamber.
Mix well contents by pipetting up and down in each well.
Transfer the cells from each well to pre-warmed buffer in 12-well plates.
Tap plate to distribute cells and put it in incubator.
Let cells recover for 24 hours.

#### Cuvette setup and electroporation

Unplug plate chamber from the power module of the Gene Pulser MXcell electroporation system and plug in the ShockPod™ cuvette chamber.
Pipette 600 μL of cell suspension into a 0.4 cm gap electroporation cuvette.
Put cuvette in ShockPod chamber and deliver electric pulse.
Remove cuvette from chamber.
Mix cuvette contents by pipetting up and down in cuvette.
Transfer the cells from each well to pre-warmed buffer in 12-well plates.
Tap plate to distribute cells and put it in incubator.
Let the cells recover for 24 hours.

### 3) Representative Results

After transfecting cells and allowing them to recover, analyze the transfection efficiency qualitatively, using epifluorescent microscopy, and quantitatively, using flow cytometry.


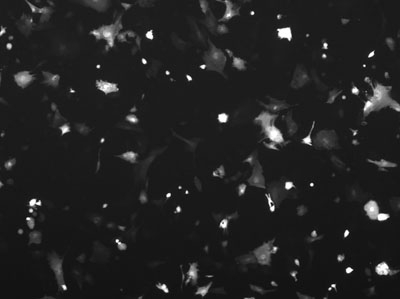
 **Figure 1.** Cells that have been successfully electroporated and are now expressing the GFP gene appear under epifluorescent  microscopy.


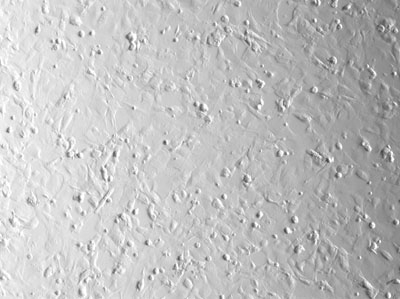
 **Figure 2.** Viewing the cells under phase contrast allows visualization of both transfected and untransfected cells.  These are the cells that were exposed to the lowest voltage electroporation pulse at 200V.  The cells are largely confluent due to the high cell density.


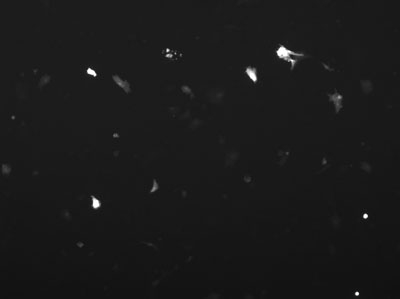
 **Figure 3.** The same field of view under epifluorescence shows a number of cells are expressing the GFP marker, but these are only a small percentage of the cells visible in the previous image.


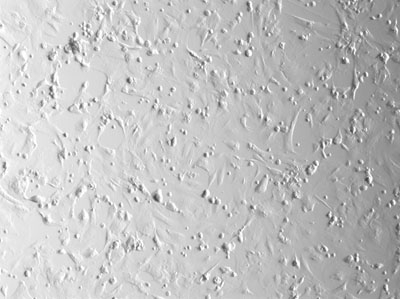
 **Figure 4.** At 250V, the total number of live cells seen under phase contrast decreases slightly.


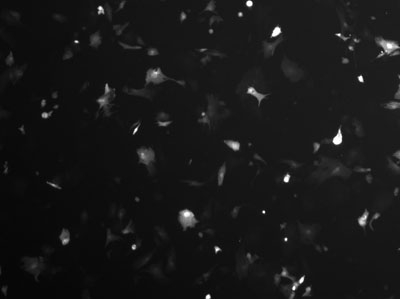
 **Figure 5.** Under epifluorescence, one can see that the number of GFP expressing cells has increased.


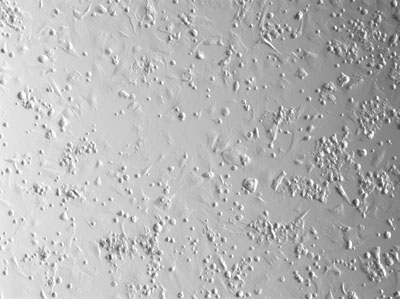
 **Figure 6.** At the highest voltage applied, 375V, there are fewer live cells visible.


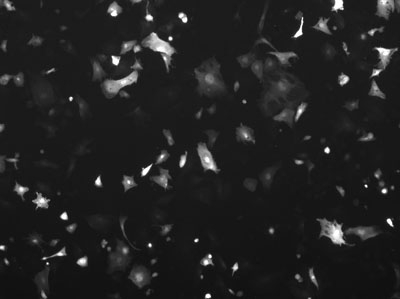
 **Figure 7.** However, a large percentage of the remaining cells are expressing GFP.  Which condition is optimal depends on the experimental design.  In some experiments the largest number of transfected cells might be optimal, in other experiments the highest percentage transfection might be best.

We are interested in the percentage of cells that are GFP positive under each condition and how the percentages vary with cell age.  Flow cytometry can provide quantitative information about the transfection results under each of the different electroporation conditions.


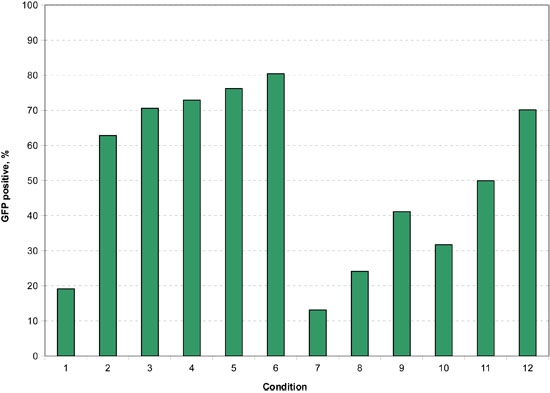
 **Figure 8.** Here the percentage of cells that are GFP positive in the passage 5 cells under each of the 12 electroporation conditions are shown.  The maximum transfection percentage was approximately 80% under the highest voltage exponential decay pulse, condition 6, and 70% under the strongest square wave pulse tested, condition 12.


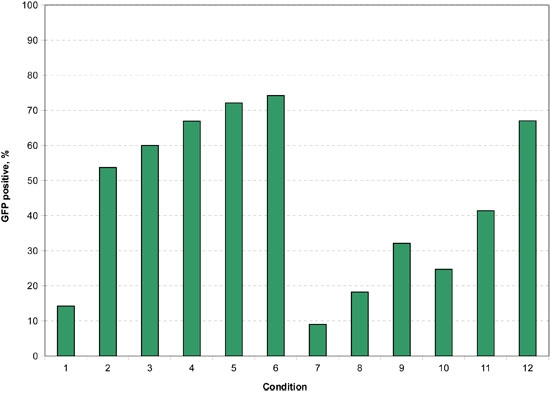
 **Figure 9.** With the cells passed 9 times prior to the electroporation, the overall pattern of transfection percentage is nearly identical, but with a very slight decrease in the transfection percentages.


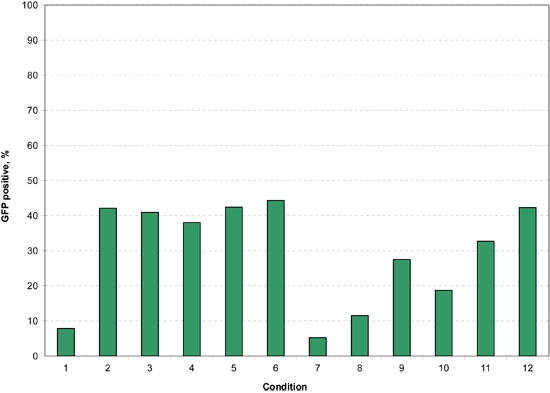
 **Figure 10.** Shown here are the percentages of GFP cells in the passage 13 cells which show a marked decrease in transfection percentage relative to the younger cells.  The highest transfection percentages were approximately half what was achieved with the younger cells demonstrating the importance of using healthy cells as soon after isolation as possible.

**Table:** 

Electroporation Conditions used for transfecting MEF cells using the Gene Pulser MXcell
Condition (1-6) Exponential Decay pulses, all with 350 uF, 1000ohm	Voltage (V)	
1	200	
2	250	
3	300	
4	326	
5	350	
6	376	
Condition (7-12) Square Wave pulses, all with 2000 uF, 1000 ohm, and 1 pulse	Voltage (V)	Pulse Duration (ms)
7	200	10
8	250	10
9	300	10
10	200	20
11	250	20
12	300	20

## Discussion

This video article demonstrates how to use the MXcell electroporation system to easily identify optimal electroporation conditions for MEFs or other primary cell lines. The 96-well plate format allows for many replicates of experimental or optimization conditions to be performed simultaneously, which can eliminate the need for many separate experiments. While carrying out this procedure, one should to remember to use healthy cells as soon after isolation as possible and to use electroporation conditions that are matched to the electroporation buffer.

